# Association of the Protein-Tyrosine Phosphatase DEP-1 with Its Substrate FLT3 Visualized by *In Situ* Proximity Ligation Assay

**DOI:** 10.1371/journal.pone.0062871

**Published:** 2013-05-01

**Authors:** Sylvia-Annette Böhmer, Irene Weibrecht, Ola Söderberg, Frank-D. Böhmer

**Affiliations:** 1 Institute of Molecular Cell Biology, Center for Molecular Biomedicine, Jena University Hospital, Jena, Germany; 2 Department of Immunology, Genetics and Pathology, Science for Life Laboratory, Rudbeck Laboratory, Uppsala University, Uppsala, Sweden; Virginia Commonwealth University, United States of America

## Abstract

Protein-tyrosine phosphatases (PTPs) are important regulators of signal transduction processes. Essential for the functional characterization of PTPs is the identification of their physiological substrates, and an important step towards this goal is the demonstration of a physical interaction. The association of PTPs with their cellular substrates is, however, often transient and difficult to detect with unmodified proteins at endogenous levels. Density-enhanced phosphatase-1 (DEP-1/PTPRJ) is a regulator of hematopoietic cell functions, and a candidate tumor suppressor. However, association of DEP-1 with any of its proposed substrates at endogenous levels has not yet been shown. We have previously obtained functional and biochemical evidence for a direct interaction of DEP-1 with the hematopoietic receptor-tyrosine kinase *Fms*-like tyrosine kinase-3 (FLT3). In the current study we have used the method of *in situ* proximity ligation assay (*in situ* PLA) to validate this interaction at endogenous levels, and to further characterize it. *In situ* PLA readily detected association of endogenous DEP-1 and FLT3 in the human acute monocytic leukemia cell line THP-1, which was enhanced by FLT3 ligand (FL) stimulation in a time-dependent manner. Association peaked between 10 and 20 min of stimulation and returned to basal levels at 30 min. This time course was similar to the time course of FLT3 autophosphorylation. FLT3 kinase inhibition and DEP-1 oxidation abrogated association. Consistent with a functional role of DEP-1-FLT3 interaction, stable knockdown of DEP-1 in THP-1 cells enhanced FL-induced ERK1/2 activation. These findings support that FLT3 is a *bona fide* substrate of DEP-1 and that interaction occurs mainly via an enzyme-substrate complex formation triggered by FLT3 ligand stimulation.

## Introduction

Protein-tyrosine phosphatases (PTPs) are important regulators of signal transduction processes [1]. While in recent years clear cellular functions for a number of PTPs could be uncovered, they are still elusive for many members of the family. A decisive issue in functional characterization of PTPs is the identification of substrates. This can be based on different experimental strategies [2]. However, the low stability of PTP-substrate complexes often precludes their isolation in immunocomplexes. Detection of PTP-substrate interactions *in situ* has so far largely relied on techniques employing engineered molecules, such as PTP-fluorescent-protein fusions in transfected cells (e.g. [3], [4]). The *in situ* proximity ligation assay (*in situ* PLA) technology allows the detection of protein-complex formation at endogenous levels, provided sufficiently specific antibodies are available [5], [6]. *In situ* PLA utilizes antibodies to which DNA oligonucleotides have been attached as probes for proximity between epitopes or primary antibodies, on fixed cells. Proximal binding of the PLA probes will allow hybridization of two additional oligonucleotides to the PLA probes. These oligonucleotides can then be ligated to form a circular DNA reporter molecule, which is amplified by rolling cycle amplification (RCA). The resulting threads of single stranded DNA will collapse into a bundle – the RCA product (RCP). RCPs are detected by fluorescence labeled oligonucleotides to generate individual bright fluorescent signals in the place where complex formation between the protein antigens was detected.

DEP-1/PTPRJ is a transmembrane PTP with high intrinsic activity, which can negatively regulate signaling of several receptor tyrosine kinases [7]–[10]. It is also a positive regulator of B-cell and macrophage immunoreceptor signaling [11], thrombocyte activation [12], and cell-matrix adhesion [13]–[15], presumably via dephosphorylation of inhibitory phosphotyrosines of Src-family tyrosine kinases. We have recently shown that DEP-1 can dephosphorylate and attenuate signaling of *Fms*-like tyrosine kinase-3 (FLT3), a hematopoietic receptor tyrosine kinase of the class III family. Based on functional analyzes, co-immunoprecipitation of FLT3 with a DEP-1 “substrate trapping” mutant, and *in vitro* dephosphorylation of immunoprecipitated FLT3 by recombinant DEP-1, we proposed a direct interaction of DEP-1 with FLT3 [16]. The importance of DEP-1-FLT3 interaction is further supported by the observation that acute myeloid leukemia cells expressing the constitutively active FLT3 ITD mutant have a compromised DEP-1 activity due to reversible oxidation, a process which contributes to cell transformation [17]. Importantly, data on complex formation of DEP-1 with FLT3 at endogenous levels have been missing up to now. Hence, in the current study we employed PLA technology to visualize complex formation of DEP-1 and FLT3 *in situ* at endogenous levels. The analysis revealed that an interaction of endogenous proteins takes place, is promoted by FLT3 ligand (FL) stimulation, and depends on FLT3 autophosphorylation.

## Materials and Methods

### Cell Lines and Reagents

COS7, THP-1, and MV4-11 cells were obtained from the German Collection of Microorganisms and Cell Cultures (DSMZ, Braunschweig, Germany). COS7 cells were cultivated in Dulbecco’s modified Eagle medium supplemented with glutamate and sodium pyruvate (PAA, Pasching, Austria) and 10% fetal calf serum (FCS; BioWest, Berlin, Germany). THP-1 cells were kept in HEPES-buffered RPMI 1640 medium, supplemented with glutamate, and 1 mM sodium pyruvate (Biochrom, Berlin, Germany) and 10% heat-inactivated FCS. MV4-11 cells were cultivated in RPMI 1640 medium, supplemented with glutamate (PAA, Kölbe, Germany), 10% heat-inactivated FCS, and 1 mM sodium pyruvate. The following antibodies were used: Rabbit polyclonal anti-FLT3 antibodies S18 (SC-480) from Santa Cruz Biotechnology (Heidelberg, Germany), polyclonal goat anti-DEP-1 (AF1934) antibodies from R&D Systems (Wiesbaden, Germany), rabbit monoclonal anti-pERK1/2 (#4695) and rabbit monoclonal anti-ERK1/2 (#4695) from Cell Signaling (Frankfurt, Germany) and FITC-labeled anti-rabbit IgG from Jackson ImmunoResearch Laboratories Inc. (West Grove, PA, USA). The selective FLT3 inhibitor cpd.102 was described earlier [18]. Poly-L-Lysine was from Sigma-Aldrich (Taufkirchen, Germany).

### DNA, siRNA and Transfections

Expression constructs for human FLT3, human DEP-1 and the corresponding C1239S mutant were described previously [19]. Transient transfection of COS7 cells was performed using polyethylenimine (PEI) as described previously [20]. DEP-1 siRNA duplex oligonucleotides [5′- UACUGUGUCUUGGAAUCUAdGdC -3′ (sense) and 5′- UAGAUUCCAAGACACAGU AdGdG -3′ (antisense)] or control siRNA (target DNA sequence AATTCTCCGAACG TGTCACGT) used in this study were obtained from Sigma-Aldrich (Taufkirchen, Germany). SiRNA was transfected into THP-1 cells using the AMAXA Nucleofector system. 1.5–2×10^6^ cells were transfected with 2.1 µg siRNA using Nucleofector buffer V and transfection program V-01 in a cuvette supplied by the manufacturer. Immediately after pulsing the suspension, cells were diluted with 0.5 ml fresh complete medium, and the mixture was transferred into a cell-culture vessel containing another 2 ml complete medium. Cells were subjected to *in situ* PLA analysis after 2 days. Stable knockdown of DEP-1 was achieved by lentiviral transduction with shRNA as described previously [15]. In brief, HEK293T cells were transfected with pLKO.1 plasmids encoding either the DEP-1-targeting shRNA or non-targeting shRNA (both in the vector pLKO.1, Sigma-Aldrich, Taufkirchen, Germany) together with the plasmids pRev, pEnv-VSV-G and pMDLg by the polyethylenimine (PEI) transfection method. 24, 48, and 72 h after transfection the medium containing the replication-deficient lentiviral particles was collected. The supernatants were concentrated 25-fold using Amicon Ultracel 30 k cartridges (Merck Millipore, Schwalbach, Germany) and 50–100 µl was used for infection of 5×10^4^ THP-1 cells (starting amount) in 1 ml medium in the presence of 8 µg/ml polybrene (1,5-dimethyl-1,5-diazaundecamethylene polymethobromide, Sigma-Aldrich, Taufkirchen, Germany) in three cycles for 16 h each on three consecutive days. Selection of stably transfected cells with 1 µg/ml puromycin (Sigma-Aldrich, Taufkirchen, Germany) was started 48 h after the third infection.

### *In situ* PLA Assays

Cells were starved in serum-free medium for 5–6 h. For treatment with the FLT3 inhibitor cpd.102, this (or DMSO for control) was added to the starvation medium (final concentration 2 µM, 0.2% DMSO). Coverslips were placed in wells of a 12-well plate and coated with poly-L-lysine (250 µg/ml in PBS) at room temperature for at least 1 h. The starved cells were sedimented and resuspended at a concentration of 1–2×10^5^ cells in 120 µl serum-free medium. This suspension was added onto a poly-L-lysine-coated coverslip and cells allowed to adhere at room temperature for 10 min. Thereafter, excess medium with non-adhered cells was aspirated, and replaced with 1.0 ml fresh, serum-free medium per well. For inhibitor treatment, this was added again at this stage. Stimulation with FLT3 ligand (FL) was done by adding 10 µl FL (final concentration 100 ng/ml) and incubation at 37°C for the appropriate length of time. Stimulation was stopped by aspirating the medium, washing once with ice-cold PBS and placing the plates on ice. To fix cells, 1 ml ice-cold 70% ethanol was added and the plates were incubated on ice for 1 h. The ethanol was aspirated and the fixed cells were air-dried at room temperature for 30 min. The plates were then immediately subjected to the staining/*in situ* PLA procedure, or stored at 4°C until use.

*In situ* PLA detection was carried out using the appropriate DUOLINK II In Situ kit components obtained from OLINK Bioscience (Uppsala, Sweden) according to the protocol of the manufacturer. In brief, cells were encircled with a wax pen, washed once with PBS, and then subjected to blocking using the DUOLINK blocking solution (1 drop) at 37°C in a wet chamber for 30 min. After two washes with Washbuffer A for 5 min each, antibodies were added at a dilution of 1∶50 in 40 µl DUOLINK antibody diluent and incubated in a wet chamber at 4°C overnight. The slides were washed two times with Washbuffer A for 5 min each, then secondary antibodies (DUOLINK anti-rabbit PLA-plus probe, DUOLINK anti-goat PLA-minus probe) were added and incubated at 37°C for 1 h. Two washes with Washbuffer A were then followed by addition of the ligation mix and incubation at 37°C for 30 min, followed by another two washes. Thereafter, the amplification reaction was carried out at 37°C for 100 min. Subsequently, the slides were washed twice with Washbuffer B, and once with 0.1×Washbuffer B. Mounting was done with mounting solution containing 1 µg/ml HOECHST 33347.

Images were acquired using an APOPTOME II Zeiss Imager Z1 microscope (Carl Zeiss Microimaging, Jena, Germany), equipped with an AXIOCAM AutoCam MR Rev3 camera and a Plan Apochromat 40 X/1.3 oil (DIC/UV VIS-IR M27) objective using Axiovision software (release 4.8.2). For each experiment, 5–10 images per condition of cell treatment were acquired using identical microscope and camera settings. Acquisition of the same image was sequentially performed in the red and blue channel (for the RCP and Hoechst staining, respectively). Corresponding TIF files were then used for quantification using BlobFinder (Version 3.2). RCP signals were detected as local maxima applying a 3×3 pixel mask. Nuclei had to have a minimum diameter of 100 pixels for detection. The procedure underlying signal recognition by the program and other image features were described in detail by Allalou *et al.* [21]. All RCP signals in the image were counted and divided by the number of nuclei to obtain an average of signals per cell in each image. Based on these data, means and standard deviations of the independently acquired images were calculated and presented.

For presentation in the figures, example images obtained in parallel in one experiment were selected. To improve visibility, contrast and brightness of the images in some figures were slightly enhanced using ImageJ 1.45 S (Wayne Rasband, National Institutes of Health, USA). For this, all images to be compared were treated identically. This procedure had, however, no effect on the quantification, which was done with the unmodified files.

### Immunoprecitipitation and Immunoblotting

Cells were starved in serum-free medium for 4 h and then stimulated with FL (100 ng/ml, 37°C) for different times as indicated in the figure legends, and then lysed with buffer containing 1% NP40, 50 mM Hepes (pH7.4), 150 mM NaCl, 1 mM EDTA, and freshly added 1 mM PMSF, aprotinin (0.1 TIU/ml), 10 µg/ml leupeptin, 2 µg/ml Pepstatin A, 1 µg/ml Pefabloc (Roche, Heidelberg, Germany), 1 mM sodium orthovanadate and PhosSTOP (Roche, Heidelberg, Germany). For analysis of FLT3 autophosphorylation, the receptor was immunoprecipitated. Cleared cell lysates (about 100 µg protein) were treated with 1.5 µg anti-FLT3 antibody overnight, followed by 20 µl protein A Sepharose beads for 2–3 h. The beads were collected by centrifugation, washed three times with buffer containing 0.1% NP40, 20 mM Hepes, pH 7.4, 150 mM NaCl, and 10% glycerol, and were subsequently extracted with SDS-PAGE sample buffer. The samples were run on 10% (acrylamide-bisacrylamide 30∶0.8, Roth, Karlsruhe, Germany) gels, alongside with pre-stained marker proteins (Page Ruler, Fermentas, St. Leon-Rot, Germany). Western blotting was performed using semi-dry transfer to polyvinylidene difluoride (PVDF) membranes at 20 V constant voltage for 60 min. The membranes were briefly washed with distilled water, then blocked in 1% BSA in 20 mM Tris pH7.6, 150 mM NaCl, 0.05% Tween 20 (TBS-T) for 1 h at room temperature (RT). Primary antibody incubation (dilution 1∶1,000) was performed in the same buffer. Antibodies detecting phosphorylation were incubated at 4°C overnight, pan-specific antibodies for 1 h at RT. After washing with TBS-T (three times at RT, 10 min each), the membranes were incubated with horseradish peroxidase (HRP)-coupled secondary antibody (anti-mouse IgG, anti-rabbit IgG, KPL, Medac, Wedel, Germany, dilution 1∶10,000; anti-goat IgG, Santa Cruz, Heidelberg, Germany, sc-2056, dilution 1∶10,000) at RT for 1 h. After washing, HRP activity was detected by enhanced chemiluminiscence using Western Lightning Plus reagent (Perkin Elmer, Rodgau-Jügesheim, Germany) and a CCD-camera based detection (LAS4000, Fujifilm, Düsseldorf, Germany) For sequential detection of phosphorylation and corresponding protein amounts, the membranes were stripped using a solution containing 100 mM β-mercaptoethanol and 2% SDS (50°C, 30 min), thoroughly washed with TBS-T, and then subjected to another detection cycle. Blots were quantified with Multi Gauge V3.0 software (Fujifilm, Düsseldorf, Germany).

### Apoptosis Detection and Proliferation Assays

The percentage of apoptotic cells was detected using an Annexin V-PE Kit (559763, BD Pharmingen, Heidelberg, Germany) with a FACSCanto flow cytometer (BD Biosciences, Heidelberg, Germany) according to the instructions of the manufacturer. Proliferation was assessed using the 3-(4,5-dimethylthiazol-2-yl)-2,5-diphenyltetrazolium bromide (MTT) method. 2×10^4^ cells were seeded per well in 96-well plates with 200 µl medium containing 0, 2, or 10% of heat-inactivated fetal bovine serum in absence or presence of 20 ng/ml FL. The MTT-conversion was measured after 48 or 72 hours as described previously [22].

### Statistic Analysis

Mean values of quantification from at least three independent experiments were subjected to analysis by one-way ANOVA (Pairwise Multiple Comparison by the Holm-Sidak method) or two-way ANOVA (as applicable) using the program Sigma Plot 12. Differences with p-values <0.05 were considered significant. Further information is provided in the figure legends.

## Results

**Figure 1:**
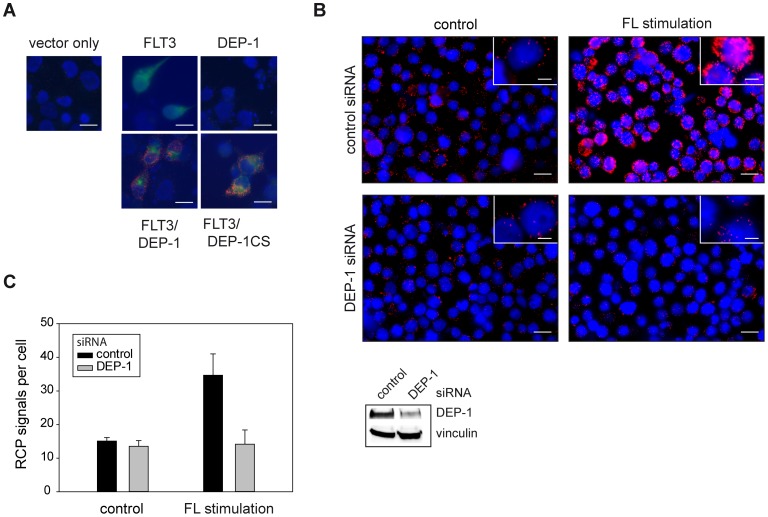
Proximity ligation assay reveals association of DEP-1 with its substrate FLT3. (**A**) COS7 cells were transiently transfected with expression constructs for FLT3, DEP-1, the catalytically inactive DEP-1 C1239S trapping mutant, or corresponding control plasmids as indicated. Complex formation was measured by *in situ* PLA using rabbit anti-FLT3 antibodies, goat anti-DEP-1 antibodies, and corresponding secondary reagents. *In situ* PLA is indicated by red signals of the rolling cycle amplification products (RCPs). FLT3 expression (green) was visualized by additional staining with FITC-labeled anti-rabbit IgG antibodies; nuclei (blue) were counterstained with Hoechst 33342. Scale bars 20 µm. (**B**), (**C**) Complex formation of endogenous DEP-1 with endogenous FLT3 in THP-1 cells. Cells were transfected with DEP-1-specific or control siRNA by Nucleofection, were then starved and either left unstimulated or were stimulated with FL (100 ng/ml) for 10 min as indicated. (**B**) Example images; DEP-1 knockdown efficiency was detected by immunblotting (lower panel). DEP-1-FLT3 complexes as RCPs are shown in red, nuclei are depicted in blue and scale bars represent 20 µm for the overview images and 5 µm for the insets. (**C**) Quantification of detected *in situ* PLA signals over 5 images per variant. The data are representative for 3 experiments with consistent results.

**Figure 2:**
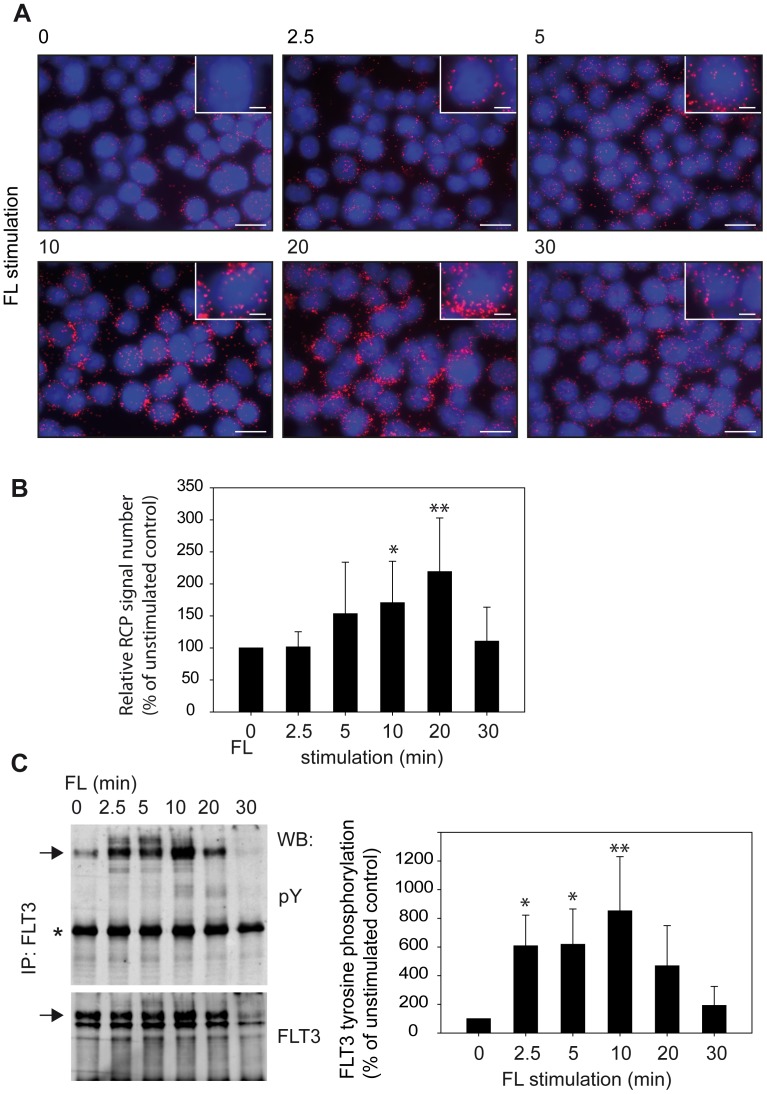
Similar kinetics of FLT3-DEP-1 complex formation and FLT3 autophosphorylation. Association of DEP-1 with FLT3 was measured by *in situ* PLA as in Figure 1. (**A**) Example images; DEP-1/FLT3 complexes as RCPs are shown in red, nuclei are depicted in blue and scale bars represent 20 µm for the overview images and 5 µm for the insets. (**B**) Cumulated data from 7 independent experiments (means ± SD, *p<0.05, **p<0.01 for difference from unstimulated sample by one-way ANOVA). (**C**) FLT3 was immunoprecipitated from THP-1 cells stimulated for the indicated timepoints and autophosphorylation was assessed by immunoblotting with anti-phosphotyrosine antibodies (pY100). FLT3 amounts were detected by reblot with anti-FLT3 antibodies. Note that for the FLT3 reblot only the section above the very abundant IgG band (star) is depicted. Two FLT3 species of 130 kDa and 150 kDa represent the immature high-mannose form and the mature, complex glycosylated form, respectively [31]. Only the 150 kDa form (arrows) becomes phosphorylated in a ligand-dependent manner. Weak bands of even higher molecular mass in the stimulated samples represent ubiquitinylated FLT3. Right panel: Quantification of multiple (n = 5) independent experiments. The pFLT3 signals were normalized to FLT3 amounts of the stripped and reprobed blots (means ± SD, *p<0.05, **p<0.01 for difference from unstimulated sample by one-way ANOVA).

**Figure 3:**
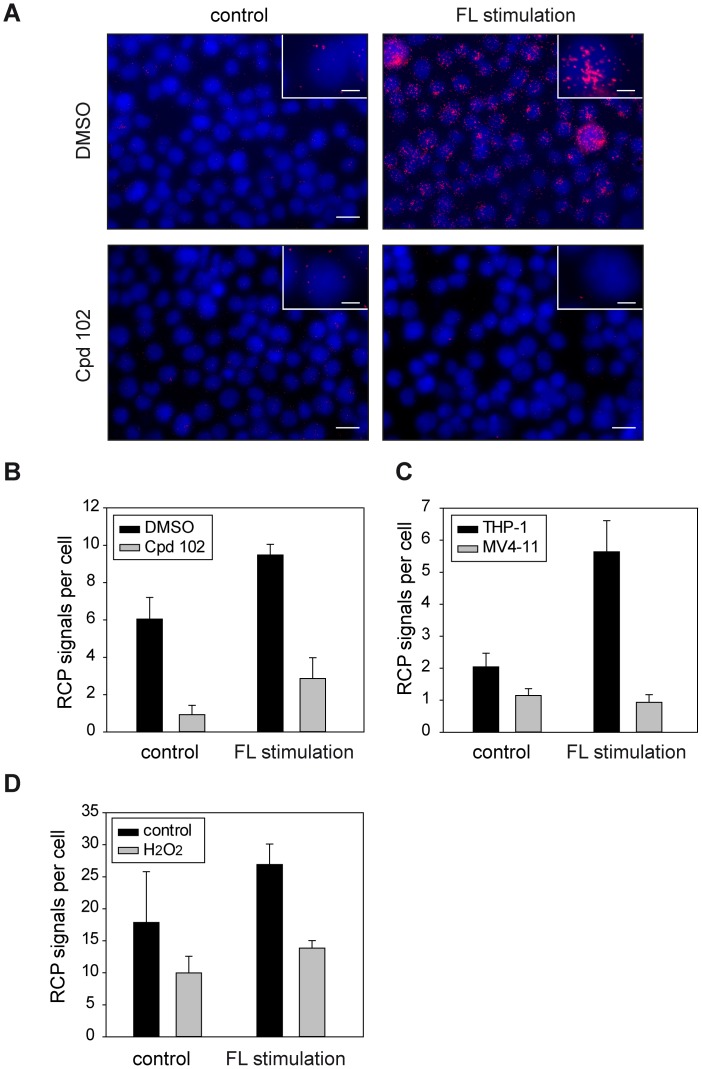
FLT3 kinase inhibition and DEP-1 oxidation interfere with complex formation. Association of DEP-1 with FLT3 was measured as in Figure 1. (**A**), (**B**) Cells were pretreated with the FLT3 kinase inhibitor compound 102 (Cpd.102, 1 µM) for 2 hours before FL stimulation (20 min). (**A**) Example images; DEP-1-FLT3 complexes as RCPs are shown in red, nuclei are depicted in blue and scale bars represent 20 µm for the overview images and 5 µm for the inset. (**B**) Quantification of 5 images per variant. (**C**) *In situ* PLA detection of complex formation between DEP-1 and FLT3 in either THP-1 cells or MV4-11 cells, harboring the oncogenic FLT3 variant FLT3 ITD. FL stimulation 20 min. (**D**) THP-1 cells were starved and pretreated with 1 mM H_2_O_2_ (5 min) before stimulation with FL (10 min) as in Figure 1 and *in situ* PLA detection. The data are representative for 3 experiments with consistent results.

To establish a suitable *in situ* PLA protocol for detection of DEP-1-FLT3 interaction, different antibodies were initially tested by conventional indirect immunofluorescence to select a pair that specifically stains the endogenous proteins in THP-1 cells (not shown). For *in situ* PLA experiments, the concentration of these antibodies was then titrated and conditions for detecting proximity were chosen similar to a previously successfully tested protocol [5] or otherwise followed the recommendations of the PLA-kit manufacturer (see Materials and Methods). To validate the selectivity of the assay we first assessed DEP-1-FLT3 complex formation in transiently transfected COS7 cells, as this system allowed performing negative controls for untransfected and single-transfected cells, as well as comparison of wildtype DEP-1 with the DEP-1 C1239S trapping mutant – the latter is known to readily form stable complexes with FLT3, which could be recovered by immunoprecipitation [16]. As shown in Figure 1A, *in situ* PLA signals were only detected in cells cotransfected with both FLT3 and DEP-1 and the number of signals was enhanced further in the case of FLT3-DEP-1 C1239S cotransfection. No signals were observed in untransfected or single-transfected cells indicating the specificity of *in situ* PLA-detection. Similar results were obtained in transfected HEK293 cells, ensuring that the assay performed well in different cell lines (not shown). Using the optimized conditions, from the experiments above, we could detect endogenous DEP-1-FLT3 complex formation in THP-1 cells under basal conditions and observed a strong increase in complex formation upon FL stimulation. No signals were detected in absence of either of the antibodies (not shown). The absolute numbers of detected RCP signals varied markedly between the individual experiments. This is likely caused by the combined variability of the multiple steps of the procedure (cell starvation and stimulation, two-step antibody binding, probe ligation, amplification by RCA followed by a highly sensitive detection) and common for PLA detection [23]. It is therefore essential to run compared samples simultaneously, which was done throughout our study. Importantly, downregulation of endogenous DEP-1 levels with siRNA greatly diminished the *in situ* PLA signal, again supporting the specificity in detection of complex formation (Fig. 1B, C). We then assessed the time-course of complex formation following ligand stimulation. The maximum of complex formation was observed at 10–20 min (Fig. 2A, B), and the levels then returned to basal values at 30 min. The time-course of FLT3 autophosphorylation was also assessed and peaked at 10 min of stimulation (Fig. 2C), somewhat earlier than the maximum of complex formation detected by *in situ* PLA. Cell treatment with the potent FLT3 kinase inhibitor compound 102 (Cpd.102) greatly diminished complex formation, both the basal and the ligand-stimulated signals (Fig. 3A, B). These observations indicated that FLT3 autophosphorylation is required to form complexes with DEP-1, consistent with an enzyme-substrate mode of association.

We also tested if oxidation of DEP-1, which is expected to abrogate substrate recognition by steric hindrance [24], [25], would affect complex formation with FLT3. To this end, we first monitored association of endogenous DEP-1 and FLT3 in the AML cell line MV4-11. While these cells express similar protein levels of DEP-1 as THP-1 cells, DEP-1 is partially oxidized and thereby inhibited by reactive oxigen species (ROS) formed downstream of the constitutively active version of FLT3– FLT3 ITD – in these cells [17]. The endogenous FLT3 ITD is, however, competent to bind DEP-1 as we have shown previously using the DEP-1 C1239S trapping mutant [16]. As expected, we could observe a greatly diminished complex formation of DEP-1 with FLT3 in MV4-11 cells, compared to THP-1 cells analyzed in parallel (Figure 3C). Moreover, treatment of THP-1 cells with hydrogen peroxide (H_2_O_2_), under conditions which are known to cause significant oxidation of DEP-1 [17], likewise diminished FLT3-DEP-1 association (Figure 3D). The reduced formation of RCPs upon H_2_O_2_ treatment was not caused by H_2_O_2_-induced apoptosis. The percentage of annexin V-positive cells was 21.2% in serum-starved cells before the experiment, and 23.1%, 18.2%, and 19.5% in cells treated with 1 mM H_2_O_2_ for 10, 20 and 30 min, respectively. Together these findings indicate that oxidation of DEP-1 prevents association with its cognate substrate FLT3.

**Figure 4:**
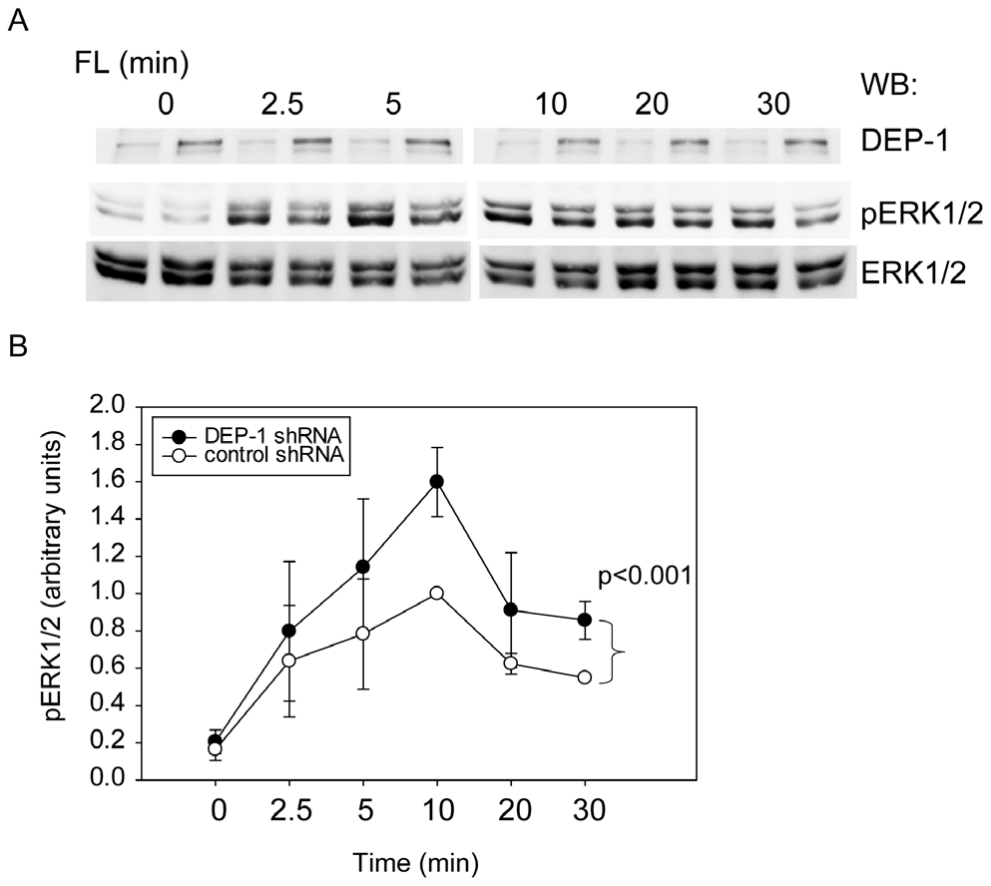
DEP-1 regulates FL-stimulated FLT3 signaling. DEP-1 expression was stably downregulated in THP-1 cells by lentiviral transduction of shRNA. Control cells harbor a non-targeting shRNA construct. (**A**) FL-dependent ERK1/2 activation was assessed by immunoblotting of cell lysate samples with antibodies recognizing activated ERK1/2 (pERK1/2). Loading was analyzed by reblot with ERK1/2 antibodies. Representative experiment; the efficiency of stable DEP-1 knockdown is also shown (upper panel). (**B**) Quantitative data for 4 independent experiments. Numbers represent pERK1/2 signals normalized to ERK1/2 levels in the same sample. The value of control cells at 10 min was set to 1.0. Significance for the difference between responses of the two different cell pools was determined by two-way ANOVA.

Previously reported experiments [16] have shown that knockdown of DEP-1 in FLT3-expressing 32D cells caused enhanced FL-induced FLT3 autophosphorylation, ERK1/2 activation, proliferation and colony formation. DEP-1 directly dephosphorylated FLT3 *in vitro*. In this previous study we have also shown that DEP-1 depletion by transient siRNA transfection caused enhanced FL-stimulated FLT3 autophosphorylation and ERK1/2 phosphorylation in THP-1 cells [16]. To confirm the functional relevance of the here shown interaction of FLT3 with DEP-1 in THP-1 cells, we generated cells with stable depletion of DEP-1 by transduction with shRNA. While FL revealed to be a poor mitogen for the THP-1 cells precluding assessment of effects of DEP-1 knockdown on cell proliferation (data not shown), survival of the DEP-1 depleted THP-1 cells in serum-free conditions was somewhat enhanced (1.26±0.18-fold over control at 48 h, assayed with 3-(4,5-dimethylthiazol-2-yl)-2,5-diphenyltetrazolium bromide (MTT), n = 5, p<0.05 by t-test). Importantly, as shown in Fig. 4A, B, FL-induced ERK1/2 activation was significantly enhanced in DEP-1 depleted cells, similarly as previously seen for the transient knockdown [16]. The latter observation further supports the functional relevance of the DEP-1-FLT3 interaction for FLT3 signaling.

## Discussion

Our *in situ* PLA experiments have indicated that formation of DEP-1-FLT3 complexes occurs at endogenous expression levels in the myeloid cell line THP-1 and that complex formation is driven by FLT3 ligand stimulation. Taken together with previous functional and biochemical studies [16], these data strongly support that FLT3 is a *bona fide* substrate of DEP-1. While proximity detected by *in situ* PLA does not allow conclusions about a direct interaction of FLT3 and DEP-1 [6], it is however likely to occur. This conclusion is supported by the dependence of complex formation on FLT3 autophosphorylation and on a reduced, active state of DEP-1. Interestingly, in the kinetic analysis the maximum of FLT3 autophosphorylation somewhat preceded the maximum of FLT3 complex formation with DEP-1 (Fig. 2C versus Fig. 2B) supporting that the former is a requirement for the latter. Moreover, the enhanced association of FLT3 with a DEP-1 trapping mutant [16], which could be recapitulated in our *in situ* PLA assays (Figure 1A), strongly argues for an efficient direct interaction [2]. Using a FRET method with transfected cells [10], ligand-enhanced association of DEP-1 to the EGF receptor has previously been shown indicating a similar mode of interaction.

While DEP-1-FLT3 association is clearly stimulated by FL, we also detected a variable basal level of association in the absence of ligand. Sensitivity of this signal to FLT3 kinase inhibition (Figure 3B), suggested that also the basal level of association depends on FLT3 autophosphorylation. The failure to suppress basal association by DEP-1 siRNA (Figure 1B, D) relates presumably to the limited efficiency of DEP-1 knockdown (Figure 1C). We therefore propose that DEP-1 is recruited to FLT3, driven by FLT3 autophosphorylation, and transiently forms an enzyme-substrate complex. Consistent with the inefficiency of co-immunoprecipitation of even highly overexpressed wildtype DEP-1 and FLT3 [16], our PLA study indicates association of DEP-1 to FLT3 through the catalytic domain. In contrast, other receptor-like PTPs may associate to their substrates through motifs outside the catalytic domain. For example, association of PTPRF/LAR with its substrates MET/HGF receptor [26] and TRK-B [27] could be readily demonstrated by co-immunoprecipitation of endogenous proteins. Different from our findings for FLT3 and DEP-1, a previous *in situ* PLA study of association of VE-PTP/PTPRB with VEGFR2 revealed a relatively high basal level of association, followed by ligand-induced complex dissociation [28]. The authors proposed that PTP dissociation may be required to enable efficient VEGFR2 phosphorylation.

Most previous studies visualizing PTP-substrate interactions have employed tagged molecules to enable FRET [3], [4], [10], BRET [29] or BiFC [30]. These analyzes were mostly carried out with cytoplasmic PTPs. Our study demonstrates the suitability of *in situ* PLA to detect the association of the receptor-like PTP DEP-1 with its substrate FLT3 at endogenous levels with native proteins. The findings support that FLT3 is a *bona fide* substrate of DEP-1, reveal specific features of DEP-1-FLT3 complex formation, and highlight the transient nature of the encounter of DEP-1 with its target. Since association of DEP-1 has not yet been shown with any of the previously proposed substrates at endogenous levels, our results present also a more general advance of knowledge with respect to this important PTP molecule.
